# Plasma neutrophil gelatinase-associated lipocalin for the prediction of acute kidney injury in acute heart failure

**DOI:** 10.1186/cc10600

**Published:** 2012-01-07

**Authors:** Tobias Breidthardt, Thenral Socrates, Beatrice Drexler, Markus Noveanu, Corinna Heinisch, Nisha Arenja, Theresia Klima, Christina Züsli, Tobias Reichlin, Mihael Potocki, Raphael Twerenbold, Jürg Steiger, Christian Mueller

**Affiliations:** 1 Department of Internal Medicine, University Hospital Basel, Peterplatz 1, Basel, 4003, Switzerland; 2 Department of Nephrology and Transplant Immunology, University Hospital Basel, Peterplatz 1, Basel, 4003, Switzerland; 3 Department of Cardiology, University Hospital Basel, Peterplatz 1, Basel, 4003, Switzerland; 4 Department of Renal Medicine, Royal Derby Hospital, Uttoxeter Road, Derby, DE22 3NE, UK; 5 Department of Nephrology, Kantonsspital, CH-5001 Aarau, Switzerland

## Abstract

**Introduction:**

The accurate prediction of acute kidney injury (AKI) in patients with acute heart failure (AHF) is an unmet clinical need. Neutrophil gelatinase-associated lipocalin (NGAL) is a novel sensitive and specific marker of AKI.

**Methods:**

A total of 207 consecutive patients presenting to the emergency department with AHF were enrolled. Plasma NGAL was measured in a blinded fashion at presentation and serially thereafter. The potential of plasma NGAL levels to predict AKI was assessed as the primary endpoint. We defined AKI according to the AKI Network classification.

**Results:**

Overall 60 patients (29%) experienced AKI. These patients were more likely to suffer from pre-existing chronic cardiac or kidney disease. At presentation, creatinine (median 140 (interquartile range (IQR), 91 to 203) umol/L versus 97 (76 to 132) umol/L, *P *< 0.01) and NGAL (114.5 (IQR, 67.1 to 201.5) ng/ml versus 74.5 (60 to 113.9) ng/ml, *P *< 0.01) levels were significantly higher in AKI compared to non-AKI patients. The prognostic accuracy for measurements obtained at presentation, as quantified by the area under the receiver operating characteristic curve was mediocre and comparable for the two markers (creatinine 0.69; 95%CI 0.59 to 0.79 versus NGAL 0.67; 95%CI 0.57 to 0.77). Serial measurements of NGAL did not further increase the prognostic accuracy for AKI. Creatinine, but not NGAL, remained an independent predictor of AKI (hazard ratio (HR) 1.12; 95%CI 1.00 to 1.25; *P *= 0.04) in multivariable regression analysis.

**Conclusions:**

Plasma NGAL levels do not adequately predict AKI in patients with AHF.

## Introduction

Acute kidney injury (AKI) is an increasingly common complication in hospitalized patients. Over the last decade the incidence of AKI has steadily increased [1]. Current estimates assume that annually about 17 million hospital admissions in the United States are complicated by AKI, resulting in additional health care costs of 10 billion dollars [2].

AKI occurring during acute heart failure (AHF) has been termed the cardiorenal syndrome Type I [3]. AHF patients developing AKI have been shown to have increased morbidity, mortality and treatment costs [4,5]. Unfortunately, the accurate prediction of AKI is an unmet clinical need [6].

Neutrophil gelatinase-associated lipocalin (NGAL; also known as human neutrophil lipocalin, lipocalin-2, siderocalin, 24p3, or LCN2) is a small molecule that belongs to the well-defined superfamily of proteins called lipocalins [7]. Human NGAL was originally identified as a 25-kDa protein associated with purified gelatinase obtained from the supernatant of activated neutrophils, which normally are the main cellular source of circulating NGAL [7]. Subsequent studies have identified tubularly secreted NGAL as a novel and specific biomarker for the early detection of AKI in several settings including cardiac surgery [8,9], contrast agent administration [10,11], unselected patients in the emergency department [12] and in critically ill patients [13-15].

As the main pathophysiological mechanisms of AKI in AHF as well as the timing of insults may differ from the settings mentioned above, it is unknown whether these promising initial findings can be extrapolated to AHF. Therefore, we aimed to examine NGAL levels in the prediction of AKI in patients with AHF.

## Materials and methods

### Setting and study population

This prospective study investigated the potential of plasma NGAL levels to predict the occurrence of AKI and worsening renal function (WRF) in consecutive AHF patients presenting to the participating emergency departments (University Hospital Basel, Kantonsspital Aarau, and Kantonsspital Luzern) from April 2006 to August 2009. To be eligible for enrollment, patients had to be over 18 years old and present with symptoms and signs of AHF. AHF was diagnosed and patients were treated according to current European Society of Cardiology (ESC) guidelines [16]. Patients undergoing chronic hemodialysis were excluded. There were no other excluding factors that may have omitted patients with renal disease or biased their enrollment in the trial. AKI and WRF diagnoses could not be determined in four patients, who were treated in the emergency department on an outpatient basis only and for whom only one serum creatinine draw and no preadmission serum creatinine values were available. These patients were excluded from the analysis. The study was carried out according to the principles of the Declaration of Helsinki. It was approved by the local ethical committee (Ethikkomission beider Basel, Reference-Number: EK 52/06). Written informed consent was obtained from all participants.

### Clinical evaluation and biomarker measurements

At the time of enrollment all patients underwent an initial clinical assessment including clinical history, physical examination, pulse oximetry, blood tests including B-type natriuretic peptide (BNP), and chest X-ray. Echocardiography, blood gas analyses and cardiac magnetic resonance imaging (MRI) were performed according to the treating physicians' recommendations. Creatinine levels were measured at presentation to the emergency department, daily for the next four days and at discharge from the hospital. Blood samples for determination of NGAL were collected at presentation, in six-hour intervals during the first 24 hours as well as after 36 hours and 48 hours. After centrifugation, samples were frozen at -80°C until assayed in a blinded fashion in a single batch using a fluorescence- immunoassay (Triage ^® ^NGAL; Alere San Diego Incorporated; San Diego, CA, USA). According to Alere the lower detection limit of the assay is 45 ng/ml, the upper detection limit is 1,310 ng/ml. The product package insert describes the lot to lot coefficient of variance as 6.4%, 5.8%, and 3.9% for low, medium, and high NGAL values, respectively. The 95th percentile in 120 healthy individuals between 18 and 83 years old was 159 ng/ml. All investigated cardiovascular drugs tested at concentrations representing at least two times the maximal therapeutic dose, did not show crossreactivity or interference with the assay.

### Endpoints

The potential of plasma NGAL levels to predict the occurrence of AKI during the first four days was assessed as the primary endpoint. We defined and graded AKI according to the Acute Kidney Injury Network (AKIN) classification as a new-onset increase in serum creatinine ≥ 0.3 mg/dl (26.4 mmol/l) over stable baseline creatinine values [17]. AKI stage 1 included acute creatinine increases between ≥ 0.3 mg/dl (≥ 26.4 μmol/l) and up to 200% over stable baseline creatinine, while increases in serum creatinine between > 200% to 300% from stable baseline were considered stage 2. AKI stage 3 entailed acute increases of serum creatinine to more than 300% (> 3-fold) as well as serum creatinine values ≥ 354 μmol/l showing an acute increase of at least 0.5 mg/dl (44 μmol/l]) [17]. We determined baseline kidney function for 189 (91%) patients by analysis of serum creatinine and medical history using the electronic medical records for the six months prior the index hospitalization. Baseline creatinine was defined as the lowest creatinine value obtained by the individual patient during the observational period. In the absence of pre-admission data the lowest creatinine value measured during the index hospitalization was accepted as the presumptive baseline value.

**Figure 1:**
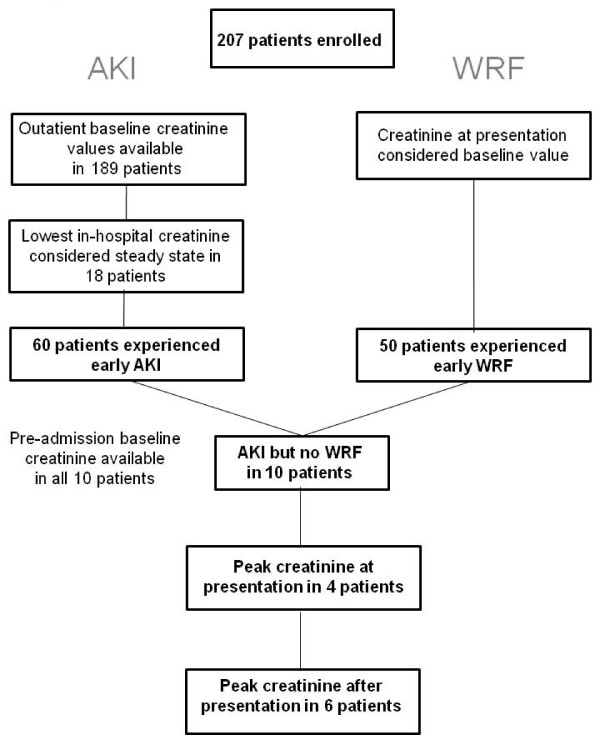
**Flow chart displaying the distribution of patients into AKI and WRF groups**.

The prediction of in-hospital WRF was considered the secondary endpoint. WRF was defined as an in-hospital increase in serum creatinine exceeding or equal to 0.3 mg/dl (26.4 mmol/l) over the value at presentation, consistent with several previous investigations [5,6]. The patient flow through this study is depicted in Figure 1.

### Statistical analysis

The statistical analyses were performed using the SPSS/PC (version 19.0,

SPSS Inc., USA) and the R-project (online at http://www.R-project.org) software packages. A statistical significance level of 0.05 was used. Discrete variables are expressed as counts (percentage) and continuous variables as means ± standard deviation (SD) or median and interquartile range [IQR], unless stated otherwise. The comparison between the two groups was done with the chi-square test and Fisher exact test for categorical variables and t-test for continuous variables if normally distributed or Mann-Whitney test if not normally distributed. The variation between the two groups of NGAL values was compared using the Kruskall-Wallis test, Mann-Whitney test and two-way analysis of variance (ANOVA) for repeated measurements. Comparisons between NGAL values at various time points for individual patients were done using Wilcoxon test. The Spearman rank correlation was used to perform correlation analyses. All hypothesis testing was two-tailed.

The prognostic accuracy of the different models was evaluated using receiver operating characteristic (ROC) curve analysis. Areas under ROC curves were compared using MedCalc software (version 9.2., MedCalc Software, Mariakerke, Belgium). Univariate and multivariate binary regression analysis was applied to identify predictors of AKI and WRF. Overall 37 candidate variables were assessed in univariate regression analysis. The variable inclusion method was used for multivariate regression analysis. Multivariate analysis included all significant candidate variables (*P *< 0.05) established in univariate analysis. To limit the effects of co-linearity between multiple variables of renal impairment a simplified multivariate analysis was also conducted. The Hosmer-Lemeshow test was conducted to compare the observed and expected frequencies of AKI and WRF based on the values of the estimated probabilities obtained by logistic regression analysis.

## Results

### Baseline characteristics

**Figure 2:**
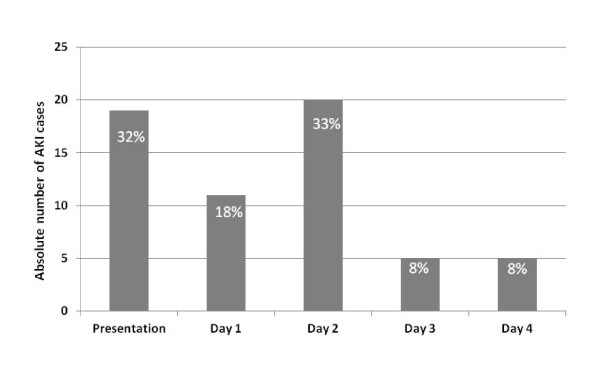
**Bar chart displaying the time of first diagnosis of AKI based on serial creatinine measurements**.

Detailed baseline characteristics of the study population are summarized in Table 1. The age of the 207 patients ranged from 39 to 98 years and the incidence of cardiovascular co-morbidities was high. Overall 60 patients experienced early AKI. These patients were more likely to suffer from a chronic cardiac or kidney disease. Additionally, AKI patients were more often treated with outpatient diuretics before being admitted to hospital and received higher doses of loop diuretics during the first three days than non-AKI patients (220 mg (85 to 328) versus 140 mg (70 to 243], *P *= 0.01). However, the more intense diuretic treatment was not associated with a larger decline in body weight or natriuretic peptide levels during the hospitalization (weight loss AKI: -3.0 kg ± 5.4 kg versus non-AKI: -4.3 kg ± 5.1 kg; *P *= 0.48; BNP change AKI: -57% (-31% to -77%) versus -48% (-8% to -70%), *P *= 0.38). The majority of AKI cases occurred within the first 48 hours, with 19 (32%) cases being present at presentation to the emergency department, 11 (18%) cases occurring within the first 24 hours and 20 (33%) cases being detected at day two (Figure 2). The majority of AKI cases were graded as AKI stage 1 (*n *= 47; 78%). Only 7 (12%) patients reached AKI stage 2 and 6 (10%) patients developed AKI stage 3.

**Table 1:** Baseline Characteristics of 207 patients

	All patients	No AKI	AKI	*P*-values
**Total number of patients**	207	147 (71)	60 (29)	
Age (year)	80 [74-85]	80 [73-84]	80 [76-86]	0.19
Female	85 (41)	59 (40)	26 (43)	0.53
**Medical History**				
Arterial Hypertension	152 (73)	106 (72)	46 (76)	0.27
Heart Failure	103 (50)	67 (46)	36 (60)	0.03
Coronary artery disease	100 (48)	66 (45)	34 (57)	0.09
Diabetes mellitus	69 (33)	49 (33)	20 (33)	0.87
Chronic kidney disease	92 (44)	57 (39)	35 (58)	< 0.01
Neoplastic disease	27 (13)	20 (14)	7 (12)	0.27
**Long term medication use**				
Diuretics	153 (74)	105 (71)	48 (80)	0.06
Betablockers	123 (59)	83 (54)	40 (66)	0.11
Nitrates	44 (21)	30 (20)	14 (23)	0.57
Renin Angiotensin System-Blockers	129 (62)	88 (61)	41 (68)	0.34
Aspirin	85 (41)	60 (41)	25 (42)	0.75
Anticoagulation	77 (37)	50 (34)	27 (45)	0.11
**Vital status**				
Blood pressure (mmHg)				
Systolic	136 [115-152]	138 [117-157]	130 [112-144]	0.05
Diastolic	84 [69-96]	84 [74-97]	75 [67-92]	0.09
Heart rate (per minute)	85 [73-102]	86 [74-100]	85 [75-107]	0.63
Respiratory rate (per minute)	20 [18-27]	20 [16-28]	22 [18-26]	0.63
Oxygen saturation (%)	97 [95-98]	96 [95-98]	97 [93-98]	0.91
Body Mass Index (kg/m^2^)	26 [23-30]	26 [23-30]	26 [24-30]	0.58
Noninvasive CVP (cmH_2_0)	13 [9-18]	13 [8-20]	13 [8-17]	0.59
**Laboratory values**				
LV ejection fraction (%)	40 [25-55]	45 [25-55]	40 [29-50]	0.87
Leucocytes (x109/L)	8.1 [6.5-10.6]	8.1[6.7-10.0]	8.2 [6.9-12.0]	0.23
Hemoglobin (g/l)	123 [114-139]	127 [120-139]	122 [107-139]	0.25
C-reactive protein (mg/l)	11.6 [4.2-32.3]	9.4 [4.1-32.9]	20.4 [8.1-40.3]	0.03
Urea (mmol/l)	9.2 [7.1-13.2]	8.9 [6.9-12.8]	11.8 [8.5-18.1]	< 0.01
Uric acid (mmol/l)	461 [370-582]	448 [347-576]	505 [407-624]	0.02
Creatinine (mmol/l)	100 [78-143]	97 [76-132]	140 [91-203]	< 0.01
Baseline eGFR (ml/min)*	52 [35-74]	57 [38-75]	42 [27-63]	< 0.01
NGAL (ng/ml)	79.6 [60.0-133.6]	75.4 [60.0-113.9]	114.5 [67.1-201.5]	< 0.01
BNP (pg/ml)	1377 [847-2428]	1321 [792-2491]	1470 [731-2444]	0.49
Troponin T (ug/l)	0.02 [0.01-0.04]	0.01 [0.01- 0.30]	0.03 [0.01-0.60]	< 0.01

Data are presented as median [interquartile range], number of patients (%)as appropriate; Abbreviations:eGFR denotes estimated glomerular filtration rate, NGAL denotes Neutrophil gelatinase-associated lipocalin; BNP denotes B-type natriuretic peptide, CVP denotes central venous pressure

### Plasma NGAL levels in patients experiencing acute kidney injury

**Figure 3:**
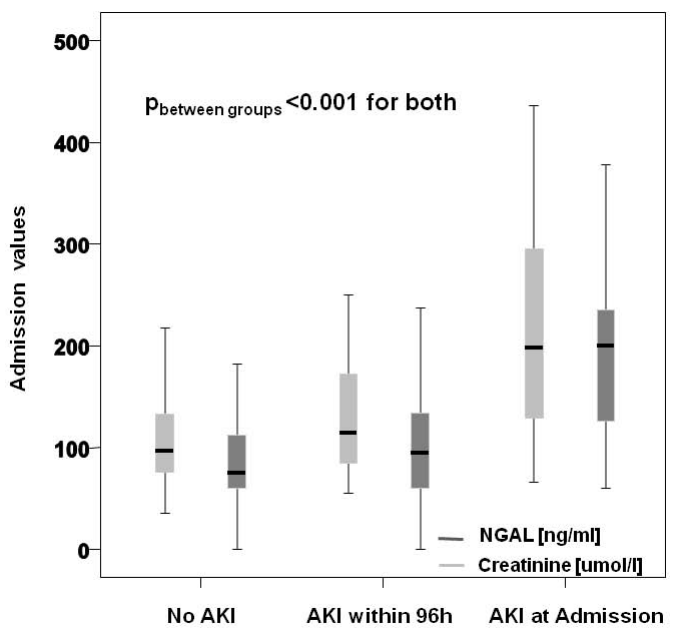
**Box Plots showing plasma NGAL levels at presentation to the emergency department in AKI and non-AKI patients**.

Plasma NGAL values at presentation of this heart failure cohort were significantly correlated with age (r = 0.27, *P *< 0.0001), admission hemoglobin levels (r = -0.23, *P *= 0.0002), admission creatinine values (r = 0.52, *P *< 0.0001) and baseline creatinine (r = 0.45, *P *< 0.0001), but not to BNP levels (r = 0.11, *P *= 0.16), leukocyte count (r = 0.15, *P *= 0.06), C-reactive protein values (r = 0.11, *P *= 0.135), body mass index (r = -0.12, *P *= 0.13) or central venous pressure (r = -0.07, *P *= 0.49) measured by non-invasive compression sonography. Using logarithmic values for non-normally distributed parameters did not change their correlations to NGAL levels at presentation. Spot measurements of plasma NGAL levels at presentation were significantly higher in patients experiencing AKI within the first four days compared to patients without AKI (114.5 ng/ml (67.1 to 201.5) versus 75.4 ng/ml (60.0 to 113.9), *P *= 0.001). Furthermore, NGAL levels were significantly associated with the severity of renal impairment (no AKI: 75.4 ng/ml (60.0 to 113.9) versus AKI stage 1: 101.8 ng/ml [(1.0 to 193.8) versus AKI stage 2: 120.9 ng/ml (60.0 to 210.7) versus AKI stage 3: 231.5 ng/ml (174.8 to 479.3); *P *< 0.001). This trend was mirrored by serum creatinine values (*P *< 0.001). NGAL levels of patients presenting with AKI were significantly higher than those of patients developing AKI during the observational time period and those not experiencing AKI at all (200.0 ng/ml (115.6 to 240.7) versus 95.6 (60.0 to 133.8) versus 74.4 (60.0 to 112.7), *P *< 0.001) (Figure 3). A similar trend could be observed for serum creatinine values.

Of note, in the 108 patients presenting with creatinine levels within the normal range (< 110 mmol/l) no differences in NGAL levels could be observed between AKI and non-AKI patients (median NGAL 60 ng/ml versus 65 ng/ml; P = 0.40).

Importantly, in an ANOVA 2-way analysis for repeated measurements neither a change of NGAL values over time (*P *= 0.66), nor a significant difference between the NGAL levels of patients experiencing AKI and those not experiencing AKI could be detected (*P *= 0.25).

### Plasma NGAL as a predictor of acute kidney injury

**Figure 4:**
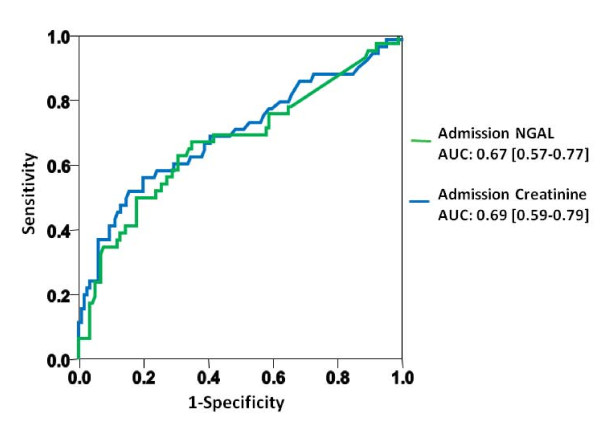
**Receiver operating characteristic curves displaying the inability of admission creatinine, and plasma NGAL to predict the occurrence of AKI**.

To evaluate the potential of serum creatinine and spot measurements of plasma NGAL at presentation to predict the occurrence of AKI, receiver operating characteristic (ROC) analyses were performed. The area under the ROC curve (AUC) for the prediction of acute kidney injury was comparable for both markers (creatinine 0.69; 95%CI 0.59 to 0.79 versus NGAL 0.67; 95%CI 0.57 to 0.77; creatinine/NGAL combined: 0.69; 95%CI 0.59 to 0.79) (Figure 4). Both models provided adequate fit (*P*-values Hosmer-Lemeshow: creatinine = 0.89; NGAL = 0.48). The calculated NGAL cut-point of 94 ng/ml that maximized the product of sensitivity times specificity achieved a sensitivity of 0.65 and a specificity of 0.65.

In patients presenting with creatinine levels within the normal range, the AUC for the prediction of AKI was poor and comparable for both markers (creatinine: 0.51 versus NGAL 0.52). Furthermore, the potential of the two markers to detect advanced AKI (stage 3) (AUC creatinine: 0.94 versus AUC NGAL 0.88) was similar.

When entering these biomarkers into a univariate binary regression analysis increasing creatinine (HR 1.13; 95%CI 1.01 to 1.20; *P *< 0.001) and NGAL levels (HR 1.01; *P *< 0.001) significantly predicted the occurrence of AKI (Table 2). During extensive multivariate regression analysis (including all candidate variables identified during univariate analysis) no marker was able to predict AKI independently. In a simplified multivariate regression analysis aimed at reducing the effects of co-linearity between multiple variables of renal impairment, admission serum creatinine remained the only independent predictor of AKI (HR 1.12; 95%CI 1.00 to 1.25; *P *= 0.04) (Table 2). Spot measurements of NGAL failed to predict the occurrence of early AKI.

**Table 2:** Prediction of the occurrence of acute kidney injury in univariate and multivariate regression analysis Univariate analysis

Predictor	Hazard Ratio	*P*-value
Age (yrs)	HR 1.03 (95%CI 0.99-1.06)	0.06
Hx Heart Failure	HR 2.02 (95%CI 1.08-3.81)	0.03
Hx Chronic Kidney Disease	HR 2.15 (95%CI 1.16-3.99)	0.02
Steady State Creatinine > 110 μol/l	HR 2.21 (95%CI 1.16-4.23)	0.02
Outpatient Diuretic Treatment	HR 2.36 (95%CI 1.03-5.41)	0.04
Systolic Blood Pressure (mmHg)	HR 0.99 (95%CI 0.97-0.99)	0.03
Loop Diuretic Dose during first 72h	HR 1.00 (95%CI 1.00-1.01)	< 0.01
Urea (mmo/l)	HR 1.09 (95%CI 1.04-1.15)	< 0.001
Uric Acid (mmol/l)	HR 1.00 (95%Ci 1.00-1.00)	0.02
C-reactive protein (mg/l)	HR 1.01 (95%CI 1.00-1.01)	0.03
Troponin T (ug/l)	HR 1.06 (95%CI 1.01-1.19)	0.02
B-type natriuretic peptide (pg/ml) (for every increase of 100 pg/ml)	HR1.00 (95%CI 0.99-1.02)	0.80
LV ejection fraction (%)	HR 0.99 (95%CI 0.97-1.02)	0.53
Creatinine (mmol/l) (for every increase of 10 mml/l)	HR 1.13 (95%CI 1.01-1.20)	< 0.001
NGAL ng/ml (for every increase of 10 ng/ml)	HR 1.01 (95%CI 1.04-1.15)	< 0.001
NGAL > 94 ng/ml	HR 1.44 (96%CI 0.15-14.20)	0.76
NGAL > 140 ng/ml	HR 3.40 (95%CI 1.61-7.15)	0.001
**Multivariate analysis**		
Hx Heart Failure	HR 1.75 (95%CI 0.64-4.80)	0.24
Steady State Creatinine > 110 μmol/l	HR 2.41 (95%CI 0.66-8.88)	0.41
Systolic Blood Pressure (mmHg)	HR 1.01 (95%CI 0.99-1.02)	0.43
Outpatient Diuretic Treatment	HR 1.25 (95%CI 0.32-4.02)	0.58
Loop Diuretic Dose during first 72h	HR 1.00 (95%CI 0.99-1.00)	0.86
C-reactive protein (mg/l)	HR 1.00 (95%CI 0.99-1.01)	0.95
Troponin T (ug/l)	HR 3.72 (95%CI 0.02-13.53)	0.63
Creatinine (mmol/l) (for every increase of 10 mml/l)	HR 1.12 (95%CI 1.00-1.24)	0.04
NGAL (ng/ml) (for every increase of 10 ng/ml)	HR 1.05 (95%CI 0.98-1.13)	0.18

To limit the effects of co-linearity between multiple parameters of renal and cardiac function multivariate analysis contained only the strongest univariate predictor in each class (acute renal function, chronic renal impairment, acute cardiac injury, chronic heart failure).

During serial sampling no significant changes in plasma NGAL levels were observed in the 41 patients experiencing AKI after presentation. Hence, NGAL changes over the first 24 hours did not provide additional predictive information.

### Plasma NGAL as a predictor of in-hospital worsening renal function

**Figure 5:**
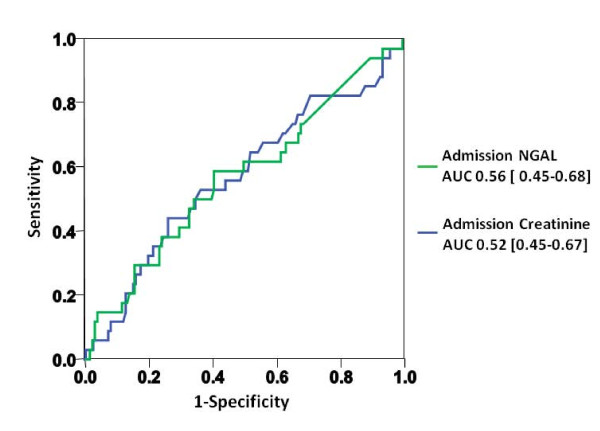
**Receiver operating characteristic curves displaying the inability of admission creatinine, and plasma NGAL to predict the occurrence of WRF**.

Overall, 50 (24%) patients experienced in-hospital WRF during the first four days. Reflecting the occurrence of WRF post presentation, admission creatinine (124 mmol/l (82 to 168) versus 110 mmol/l (82-150), p = 0.80) and plasma NGAL (94.0 ng/ml (60.0-182.4) versus 83.7 (0.0 to 135.3), *P *= 0.34) levels were similar in WRF and non-WRF patients. Again, no significant change over time (*P *= 0.46) or inter-group differences (*P *= 0.43) were found during repeated measurements. In ROC curve analysis the AUC was comparable for both markers (creatinine: 0.56; 95%CI 0.45 to 0.67 versus NGAL: 0.52; 95%CI 0.41 to 0.63) (Figure 5). In a univariate regression analysis model NGAL values (HR 1.00; 95%CI 0.99 to 1.00; *P *= 0.09) failed to predict WRF independently.

## Discussion

In this investigation we examined the potential of plasma NGAL levels at admission and serially thereafter to predict the occurrence of AKI and WRF in 207 patients with AHF. There are several key findings in this study. First, in AHF patients, plasma NGAL levels are significantly correlated to renal indices at presentation but also to age and baseline renal function. However, plasma NGAL levels are not associated with the extent of hemodynamic cardiac stress as quantified by BNP, the extent of venous congestion or inflammation. Second and most importantly, we found that spot measurements of plasma NGAL at presentation failed to improve the prediction of AKI over creatinine measurement. While both markers were significantly higher in patients experiencing AKI, their accuracy for the prediction of AKI was only modest. Third, the combination of plasma NGAL levels and creatinine did not result in a higher accuracy than creatinine alone. Fourth, the accuracy of plasma NGAL was even lower in patients presenting with creatinine values within the normal range. Fifth, plasma NGAL levels did not show a clear trend preceding the occurrence of AKI. Consequently, serial measurements did not add to the diagnostic potential of plasma NGAL at presentation.

Our results represent the first larger scale study investigating the potential of plasma NGAL in the setting of AHF. The results of an earlier study enrolling 91 patients with AHF support our observations. Aghel and colleagues also found spot measurements of serum NGAL to be significantly higher in patients experiencing early AKI [18]. Furthermore, their study described comparable AUCs for serum NGAL and estimated glomerular filtration rate (eGFR) for the prediction of early AKI (0.7 and 0.61, respectively). Unfortunately, due to the small number of patients in their study no extensive multivariate analysis could be performed. Contrastingly, a very recent study by Alvelos and co-workers found spot measurements of NGAL to powerfully predict AKI in 114 hospitalized AHF patients (AUC 0.93)[19]. However, their study differs significantly from the present study and also the work presented by Aghel, since it selectively assessed the predictive potential of NGAL levels drawn on the first in-hospital day and only considered AKI occurring between day two and day three. Applying these criteria to our population led to the exclusion of 24 (40%) early AKI cases, explaining the lower incidence of AKI occurring in the Alvelos study (12% versus 29%). Additionally, using the Alvelos definition of early AKI in a secondary analysis, we found the predictive accuracy of NGAL levels at presentation to be comparable to our primary analysis (AUC 0.61) and were therefore not able to confirm their findings. It can only be speculated that the narrower and sub-acute observational period in the Alvelos study reflects a more specific, NGAL-detectable AKI pathology, while the more inclusive observational period in this study might cover a wider range of AKI triggers.

The results obtained for AHF patients differ from the results obtained in other clinical settings, such as after cardiac surgery [8,9] and contrast agent administration [10,11], in which NGAL was established as a promising early marker of AKI. However, contrast-induced nephropathy and post-surgical AKI are marked by a clear time of injury. In these settings NGAL has repeatedly been proven to increase significantly three to four hours after the insult, while creatinine only increases 48 to 72 hours later. In contrast, the pathophysiological mechanisms underlying renal dysfunction in patients with AHF appear to be multifactorial and not well defined. Overall, an imbalance between the failing heart, the neurohumoral system and inflammatory responses has been associated with the occurrence of renal dysfunction [20]. Additionally, hemodynamic factors including the adequacy of renal perfusion [21] and the degree of venous congestion [22,23] as well as drug nephrotoxicity appear to contribute to the development of AKI in AHF. Hence, AKI in AHF might well be caused by serial insults making its prediction especially difficult. Similarly, two studies directly comparing the predictive potential of admission NGAL to admission creatinine [13] or clinical prediction models [15] in critically ill patients, found classical predictors of AKI to at least equal if not surpass the potential of NGAL. Hence, even in the setting of critically ill patients, NGAL levels might not be able to act as a stand-alone test [24,25].

The significant correlation of admission NGAL levels with baseline creatinine levels observed in this study and the correlation of urinary NGAL levels with renal indices in stable chronic heart failure [26,27] suggest that NGAL levels in AHF may reflect chronic structural tubular damage. This thesis is further supported by the stable NGAL levels over time in patients experiencing AKI in this study. A chronic structural tubular damage and persistently elevated NGAL levels appear to be widespread in the setting of heart failure [26] and elevated NGAL levels have been associated with increased short-term mortality [28,29]. Consequently, the dampened kinetics of NGAL levels before the occurrence of AKI might contribute to the limited predictive potential of plasma NGAL in AHF.

Additionally, recent studies found circulating NGAL levels to be influenced by various extra-renal factors [30]. Systemically elevated NGAL levels have been observed in infectious diseases [7], primary and secondary anemia [31], various human cancers [30] and animal models of obesity [32]. In the setting of cardiovascular diseases, elevated NGAL levels were found in coronary plaques, the failing myocardium and in the circulation of heart failure patients [33]. The combined influence of this systemic NGAL pool might contribute to the limited predictive potential of plasma NGAL in AHF. The predictive potential of urinary NGAL, which appears to be less impacted by extra-renal factors, has not been selectively examined in AHF patients yet. However, a recent meta-analysis including patients from 19 studies showed comparable performances for plasma and urinary NGAL [34].

Potential limitations of the current study merit consideration. First, only Swiss centers participated in this study. However as baseline characteristics, treatment and the frequency of AKI rates were similar to those observed in large registry studies, we consider our results representative. Second, we did not include the urine output criteria of the AKIN classification into the definition and grading of AKI, as loop diuretics impacting urine were used in the majority of our patients and adequate urine output collection is especially difficult during the early in-hospital period marked by repeated changes of the care-team. However, these difficulties are shared by previously published studies assessing the predictive potential of NGAL in the non-ICU setting, which also opted to exclude the urine output criteria for AKI diagnosis [12,18,19]. Third, we cannot differentiate between 'pre-renal' and 'structural" disturbances of kidney function as the reason for AKI. However, independent of the underlying pathology, minor changes of renal function have repeatedly been associated with a worse patient outcome [35,36]. We therefore believe that adequate prediction of AKI in AHF should be able to foresee these prognostically important creatinine changes. Fourth, the definition of AKI is based on serial measurements of serum creatinine, hence an incorporation bias [37,38] might have favored presentation values of serum creatinine in predicting AKI in ROC curve and regression analyses. However, in the absence of a non-creatinine based definition of AKI, we feel that to provide tangible clinical benefit plasma NGAL would have to improve the early detection of AKI over the current gold standard. Finally, clinical parameters (most importantly chronic kidney disease) previously shown to predict in-hospital AKI (observational period presentation to discharge) [6,39,40] failed to independently predict early AKI in this study. These differences most likely represent the increased importance of acute parameters (serum creatinine incorporating community acquired AKI and chronic kidney disease) versus chronic parameters (chronic kidney disease, outpatient diuretic therapy) in the prediction of early versus late AKI. Nevertheless, these clinical parameters remained non-significantly associated with an increased occurrence of early AKI.

## Conclusion

Plasma NGAL levels do not adequately predict AKI and WRF in patients with AHF. This lack of predictive power is probably caused by the multifactorial pathomechanism underlying the cardiorenal syndrome and chronically elevated NGAL levels caused by chronic tubular injury and extrarenal NGAL production in heart failure patients.

## Key messages

• Spot measurements of plasma NGAL at presentation do not improve the prediction of AKI over creatinine in patients with acute heart failure.

• The combination of plasma NGAL levels and creatinine does not result in a higher predictive accuracy than creatinine alone.

• In acute heart failure plasma NGAL levels do not show a clear trend preceding the occurrence of AKI.

• Consequently, serial measurements do not add to the predictive potential of plasma NGAL at presentation.

## Abbreviations

AHF: acute heart failure; AKI: acute kidney injury; AKIN: Acute Kidney Injury Network; AUC: area under the curve; ANOVA: analysis of variance; BNP: B-type natriuretic peptide; eGFR: estimated glomerular filtration rate; IQR: interquartile range; MRI: magnetic resonance imaging; NGAL: neutrophil gelatinase-associated lipocalin; ROC: receiver operating characteristic; WRF: worsening renal function

## Competing interests

CM has received research support from Abbott, Alere, Biosite, Brahms, Roche, Siemens and Behring. The NGAL measurements were provided free of charge by Alere. The other co-authors have no competing interests.

## Authors' contributions

TB and CM participated in the study concept and design, acquisition of data, analysis and interpretation of data, drafting of the manuscript, critical revision of the manuscript for important intellectual content and had full access to all of the data in the study and take responsibility for the integrity of the data and the accuracy of the data analysis. TS, BD, MN, TK, NA, CZ, CH, TR, MP, and RT participated in acquisition of data, analysis and interpretation of data and critical revision of the manuscript for important intellectual content. JS participated in analysis, interpretation of data, drafting of the manuscript and critical revision of the manuscript for important intellectual content. All authors read and approved the final manuscript.
